# Nomogram models for predicting sarcopenia in elderly Asian patients with type 2 diabetes

**DOI:** 10.1016/j.clinsp.2025.100771

**Published:** 2025-09-06

**Authors:** Hong Zhang, Ying Jin, Shan Che, Zhen Song

**Affiliations:** a Department of Internal Medicine, Jinan Second Maternal and Child Health Hospital, Laiwu District, Jinan City, Shandong Province, China; b Ultrasound Department, Jinan People's Hospital, Laiwu District, Jinan City, Shandong Province, China

**Keywords:** Sarcopenia, Type 2 Diabetes Mellitus, Risk factors, Nomogram, Predictive Modeling

## Abstract

*Background*: Sarcopenia is a prevalent but underrecognized complication in elderly patients with Type 2 Diabetes Mellitus (T2DM). Its complex etiology limits early diagnosis and intervention. This study developed and internally validated a nomogram for individualized sarcopenia risk assessment in this population.

*Methods*: A cross-sectional study was conducted involving 300 elderly patients diagnosed with T2DM from two tertiary medical centers. Sarcopenia was identified based on the Asian Working Group for Sarcopenia (AWGS) criteria, incorporating both dual-energy X-Ray Absorptiometry (DXA) measurements and handgrip strength assessments. Potential risk predictors were initially filtered using the Least Absolute Shrinkage and Selection Operator (LASSO) method, followed by multivariable logistic regression to ascertain independent factors. A nomogram model was then established integrating these variables. Model performance was assessed through Receiver Operating Characteristic (ROC) curve analysis, calibration plots, and Decision Curve Analysis (DCA) for clinical applicability.

*Results*: Sarcopenia was present in 22 % of participants. Independent predictors included age, sex, glycosylated Hemoglobin (HbA1c), serum albumin, thyroid dysfunction, and physical activity level. The nomogram demonstrated strong predictive accuracy, with an area under the ROC curve of 0.891 in the development cohort and 0.868 in the validation cohort. Calibration and decision curve analyses confirmed its reliability and clinical utility.

*Conclusions*: The proposed nomogram enables early identification of sarcopenia risk in elderly patients with T2DM, supporting personalized intervention strategies. Prospective validation in diverse populations is warranted to enhance its generalizability.

## HIGHLIGHTS

• Identified key sarcopenia risk factors in elderly patients with type 2 diabetes.
• Developed a predictive nomogram using clinical and lifestyle-based variables.
• Model showed high accuracy with AUCs of 0.891 and 0.868 in two cohorts.
• Validated nomogram supports early sarcopenia screening in diabetic patients.

## Introduction

Sarcopenia, a condition marked by the progressive decline in skeletal muscle mass, strength, and functionality, has gained recognition as a significant public health concern, particularly among elderly populations.^1^, ^2^, ^3^ Among elderly patients with T2DM, the prevalence of sarcopenia is notably higher, attributed to the combined effects of chronic hyperglycemia, insulin resistance, and systemic inflammation.^4^, ^5^, ^6^ This dual burden increases the risk of frailty, falls, and disability, leading to higher healthcare costs and diminished quality of life.^7^

Despite increasing awareness of the clinical impact of sarcopenia, its multifactorial etiology in elderly T2DM patients remains underexplored.^8^ Current evidence suggests that aging, glycemic control, nutritional status, and thyroid dysfunction are associated with sarcopenia development.^9^, ^10^, ^11^ However, most existing studies focus on isolated risk factors, overlooking the cumulative effect of multiple variables on sarcopenia risk. A comprehensive approach to identify and quantify these risk factors is crucial for early identification and intervention.

Given the growing clinical relevance of sarcopenia, there is an urgent need for a robust predictive model to estimate the individual risk of sarcopenia in elderly T2DM patients.^12^ Such a model could support personalized intervention strategies, improve clinical outcomes, and reduce healthcare costs. Addressing this gap, the present study aims to identify key predictors of sarcopenia and develop a predictive nomogram integrating demographic, clinical, and lifestyle factors. By providing a comprehensive analysis of these factors, the authors hope to identify actionable targets for mitigating the impact of sarcopenia and improving health outcomes in this high-risk population.

## Methods

### Study design and participants

This study followed the Strengthening the Reporting of Observational Studies in Epidemiology (STROBE) Statement guidelines for cross-sectional observational studies. It aimed to identify risk factors associated with sarcopenia in elderly patients with T2DM. Participants were recruited from two tertiary care hospitals in Jinan, China, between January and December 2023, using convenience sampling. Eligible patients attending the diabetes or internal medicine clinics during this period were approached for participation. Inclusion criteria required participants to be aged 60 to 80 years with a confirmed diagnosis of T2DM based on ADA criteria.^13^ Patients with incomplete records, prior sarcopenia diagnoses, or coexisting conditions that independently affect muscle mass (e.g., advanced cancer, end-stage renal disease) were excluded. Detailed medical histories were reviewed to ensure eligibility and reduce selection bias.

To ensure the study's representativeness, participants were stratified by age and gender, capturing a broad range of clinical and demographic profiles. While convenience sampling was employed to address feasibility constraints, efforts were made to minimize selection bias and ensure a diverse sample, providing a reliable foundation for subsequent analyses.

### Ethical considerations

This study was conducted in accordance with the principles of the Declaration of Helsinki. The Ethics Review Board of Jinan Second Maternal and Child Health Hospital approved the study (IRB approval number: LWFY-2023–015). As the study involved a retrospective analysis of existing medical records, informed consent was not required. All participant data were anonymized to ensure confidentiality and stored securely in compliance with data protection regulations.

### Sarcopenia diagnosis

Sarcopenia was diagnosed according to the criteria established by the Asian Working Group for Sarcopenia (AWGS).^14^ Muscle mass was assessed using Dual-Energy X-Ray Absorptiometry (DXA), with an Appendicular Skeletal Muscle Mass Index (ASMI) of < 7.0 kg/m^2^ for men and < 5.4 kg/m^2^ for women. Muscle strength was measured using a digital hand dynamometer (Jamar Hydraulic Dynamometer, Model 5030J1). Participants were seated with the elbow at a 90-degree angle and the forearm in a neutral position. Both hands were tested three times, with a 1-minute rest between trials. The highest value from either hand was recorded as the handgrip strength. Cut-off values for sarcopenia diagnosis were < 28 kg for men and < 18 kg for women, as per AWGS guidelines.

### Data collection

To ensure consistency in data collection, a structured protocol was implemented, covering a comprehensive range of demographic, clinical, and lifestyle variables. Demographic data included age and gender, both of which are critical determinants of sarcopenia risk. Clinical measures included the duration of diabetes, glycemic control as indicated by HbA1c levels, serum albumin as a marker of nutritional status, vitamin D levels, thyroid function, and the presence of hyperlipidemia.

Lifestyle variables were assessed using validated tools and structured interviews. Alcohol consumption and smoking history were assessed through self-reported questionnaires. Physical activity was measured using the International Physical Activity Questionnaire (IPAQ), which has been culturally adapted and validated for older Chinese populations. Activity levels were categorized as low, moderate, or high based on metabolic equivalent scores derived from self-reported data.

### Statistical analysis

Continuous variables were evaluated for normality using histograms and the Shapiro-Wilk test. Normally distributed data were summarized as means ± SD, while skewed data were presented as medians with Interquartile Ranges (IQR). Categorical variables were presented as frequencies and percentages. To identify the most informative predictors, Least Absolute Shrinkage and Selection Operator (LASSO) regression was applied, with the optimal tuning parameter (λ) determined via 10-fold cross-validation. This method ensured robust predictor selection while minimizing the risk of overfitting.

A multivariate logistic regression model was subsequently developed using the predictors identified by LASSO regression to assess their associations with sarcopenia risk. Odds Ratios (ORs) with 95 % Confidence Intervals (95 % CIs) were calculated to quantify the strength and direction of these associations. The model's performance was assessed using the Area Under the Receiver Operating Characteristic Curve (AUC-ROC), and calibration was evaluated using calibration plots. To enhance clinical applicability, a nomogram was constructed based on the logistic regression model. The nomogram was validated using an independent test cohort to assess its accuracy and generalizability. All statistical analyses, including nomogram construction and validation, were performed using *R* software (version 4.4.2), with a two-tailed p-value of < 0.05 considered statistically significant.

## Results

### Baseline characteristics

A total of 300 elderly patients with T2DM were enrolled in this study, of whom 66 (22.0 %) were diagnosed with sarcopenia. The baseline characteristics of the participants stratified by sarcopenia status are presented in Table 1. Patients with sarcopenia were significantly older (mean age: 71.5 ± 4.8 years vs. 69.6 ± 5.0 years, *p* = 0.006) and predominantly male (71.2 % vs. 28.8 %, *p* = 0.007). They also demonstrated poorer glycemic control, evidenced by higher mean HbA1c levels (8.59 % ± 0.73 % vs. 7.72 % ± 0.95 %, *p* < 0.001) and lower serum albumin concentrations (3.74 ± 0.37 g/dL vs. 3.93 ± 0.38 g/dL, *p* < 0.001). Additionally, vitamin D levels were significantly reduced in the sarcopenia group (20.54 ± 4.28 ng/mL vs. 22.20 ± 5.52 ng/mL, *p* = 0.027). The prevalence of comorbidities such as hyperlipidemia, hypothyroidism, and low physical activity was significantly higher among participants with sarcopenia (*p* < 0.05 for all). Furthermore, analysis of physical activity levels by gender revealed that among male participants, 42.9 % engaged in low physical activity, 27.1 % in moderate, and 30.0 % in high levels. In contrast, among female participants, 35.4 % reported low physical activity, 22.3 % moderate, and 42.3 % high (χ^2^ = 4.88, *p* = 0.037).

**Table 1:** Gender-specific prevalence of sarcopenia and its association with clinical variables.

Characteristic	Overall (*n* = 300)	No Sarcopenia (*n* = 234)	Sarcopenia (*n* = 66)	p-value^a^
Age (years), Mean (SD)	70.04 (5.01)	69.63 (4.99)	71.50 (4.81)	0.006
Gender, n ( %)				0.005*
Male	170 (56.67 %)	123 (52.56 %)	47 (71.21 %)	
Female	130 (43.33 %)	111 (47.43 %)	19 (28.88 %)	
Body Mass Index (kg/m^2^), Mean (SD)	27.16 (3.20)	27.12 (3.28)	27.28 (2.93)	0.764
Duration of Diabetes (years), Mean (SD)	11.61 (3.10)	11.20 (3.11)	13.08 (2.62)	<0.001**
HbA1c ( %), Mean (SD)	7.91 (0.98)	7.72 (0.95)	8.59 (0.73)	<0.001**
Serum Albumin (g/dL), Mean (SD)	3.89 (0.38)	3.93 (0.38)	3.74 (0.37)	<0.001**
Vitamin D (ng/mL), Mean (SD)	21.83 (5.31)	22.20 (5.52)	20.54 (4.28)	0.027*
Hypertension, n ( %)	196 (65.33 %)	154 (65.81 %)	42 (63.64 %)	0.743
Hyperlipidemia, n ( %)	171 (57.19 %)	126 (54.08 %)	45 (68.18 %)	0.041*
Hypothyroidism, n ( %)	70 (23.33 %)	48 (20.51 %)	22 (33.33 %)	0.030*
Smoking History, n ( %)	122 (40.67 %)	93 (39.74 %)	29 (43.94 %)	0.540
Alcohol History, n ( %)	103 (34.33 %)	71 (30.34 %)	32 (48.48 %)	0.006*
Physical Activity Level, n ( %)				0.004*
Low	116 (38.67 %)	79 (33.76 %)	37.00 (56.06 %)	
Moderate	138 (46.00 %)	115 (49.15 %)	23.00 (34.85 %)	
High	46 (15.33 %)	40 (17.09 %)	6.00 (9.09 %)	

a Pearson’s χ^2^ test for categorical variables; Wilcoxon rank sum test for continuous variables.

### Variable selection using lasso regression

**Fig. 1:**
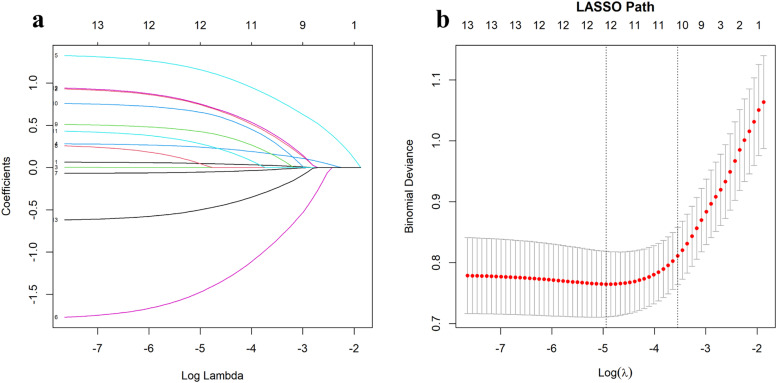
LASSO Regression for Variable Selection. (a) Coefficient profiles for candidate variables as the penalty (log Lambda) changes. Each curve represents a variable, with the optimal tuning parameter selected using 10-fold cross-validation. (b) Binomial deviance across log Lambda values. The dotted vertical lines indicate the optimal value (λ = 0.0288) used to identify predictors with non-zero coefficients.

To identify the most significant predictors of sarcopenia, LASSO regression was applied to the development cohort. The optimal tuning parameter (λ) was determined using 10-fold cross-validation, yielding a λ value of 0.0288 (λ.1se). This process identified ten variables with non-zero coefficients as the most predictive features: age, gender, duration of diabetes, HbA1c (glycated hemoglobin), serum albumin, vitamin D, hyperlipidemia, hypothyroidism, alcohol history, and physical activity level. The coefficient profiles and binomial deviance used to select the optimal λ value are shown in Fig. 1.

These variables were considered the most informative factors associated with sarcopenia risk in the study population and were subsequently incorporated into a multivariate logistic regression model to develop a predictive nomogram.

### Model development and validation

A multivariate logistic regression model was constructed using the development cohort and based on the ten variables selected through LASSO regression. The results of the logistic regression analysis are summarized in Table 2, including the odds ratios, 95 % Confidence Intervals, and p-values for each predictor.

**Table 2:** Multivariate logistic regression analysis for sarcopenia risk factors.

Characteristic	OR (95 % CI)	p-value
Age	1.06 (0.99, 1.14)	0.092
Gender (Male)	2.52 (1.17, 5.43)	0.018^a^
Duration of Diabetes	1.33 (1.16, 1.52)	<0.001**
HbA1c	3.78 (2.44, 5.88)	<0.001**
Serum Albumin	0.18 (0.07, 0.48)	0.001**
Vitamin D	0.93 (0.86, 1.01)	0.072
Hyperlipidemia	1.69 (0.80, 3.57)	0.169
Hypothyroidism	2.41 (1.07, 5.39)	0.033^a^
Alcohol History	2.53 (1.20, 5.33)	0.014^a^
Physical Activity Level	0.54 (0.30, 0.97)	0.039^a^

Odds Ratio (OR): The odds of sarcopenia associated with a one-unit increase in the predictor. 95 % CI, Confidence Interval for the OR. Significance levels: *p* < 0.05^a^.

**Fig. 2:**
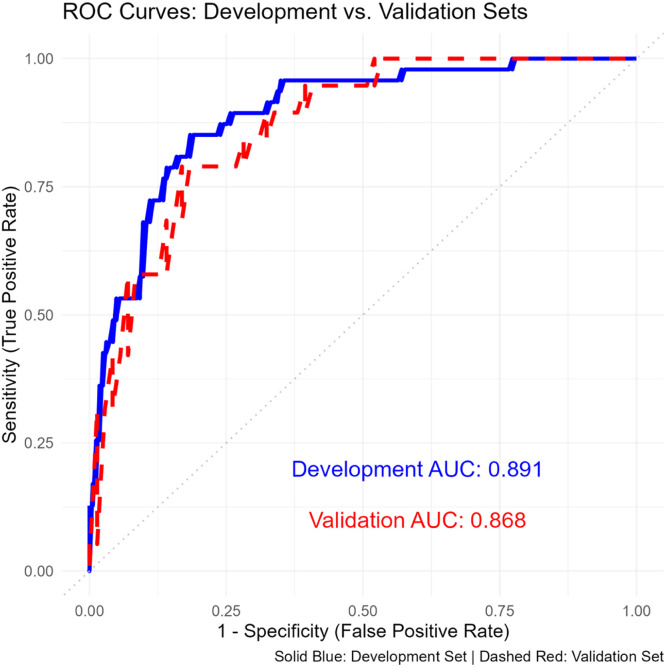
Nomogram for predicting risk of sarcopenia.

To enhance interpretability, a nomogram was developed to visualize the logistic regression model. This tool provides clinicians with an intuitive, user-friendly method for estimating an individual patient’s probability of sarcopenia by integrating the contributions of multiple predictors. Each predictor’s relative importance is represented on a scoring scale, allowing for personalized risk stratification and informed clinical decision-making. The nomogram, shown in Fig. 2, highlights key predictors such as age, gender, HbA1c, serum albumin, and others. By calculating a cumulative score, clinicians can efficiently determine a patient’s probability of sarcopenia and tailor intervention strategies accordingly.

A nomogram constructed from the predictive model incorporates key variables such as age, gender, HbA1c, serum albumin, thyroid function, and physical activity level. Clinicians can calculate a total score to estimate an individual's risk of sarcopenia, facilitating personalized risk assessment.

### Model discrimination

**Fig. 3:**
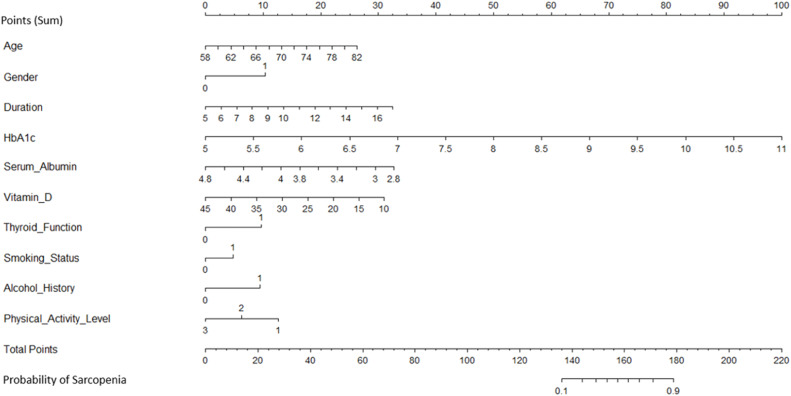
ROC Curves for the development and validation cohorts.

The model's discrimination ability was evaluated using the ROC curve and the AUC. The nomogram demonstrated strong predictive accuracy, with an area under the ROC curve of 0.891 in the development cohort and 0.868 in the validation cohort. The ROC curves for both the development and validation cohorts are shown in Fig. 3.

ROC curves illustrating the discriminative ability of the predictive model. The development cohort achieved an AUC of 0.891 (blue), and the validation cohort achieved an AUC of 0.868 (red). These results demonstrate excellent model discrimination in both datasets.

DeLong's test for two correlated ROC curves revealed no significant difference between the AUCs of the development and validation cohorts (*p* = 0.645). This consistency underscores the model’s reliability and robustness across different patient groups.

### Calibration curve evaluation

**Fig. 4:**
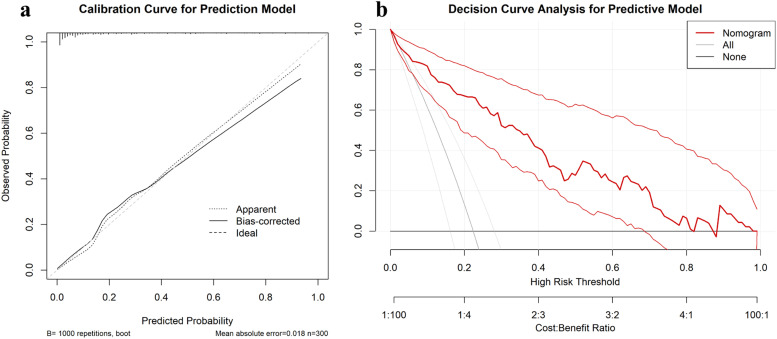
Calibration and Decision Curve Analysis of the Predictive Model. (a) Calibration curve comparing predicted probabilities to observed outcomes, demonstrating excellent agreement with a mean absolute error of 0.018. (b) DCA showing significant net clinical benefit for the nomogram compared to “treat all” and “treat none” strategies across a range of threshold probabilities.

The calibration of the predictive model was assessed using calibration curves, which compare predicted probabilities with observed outcomes. The bias-corrected calibration curve closely followed the ideal diagonal line (Fig. 4a), indicating excellent agreement between predicted and actual probabilities. The Mean Absolute Error (MAE) of 0.018, calculated from 1000 bootstrap repetitions, further confirmed the model’s robustness and calibration accuracy.

### Decision curve analysis

The clinical utility of the model was evaluated using Decision Curve Analysis (DCA). As shown in Fig. 4b, the DCA curve demonstrated a significant net benefit across a range of threshold probabilities (approximately 0.1 to 0.8), compared to the “treat all” and “treat none” strategies. This finding highlights the nomogram's potential to guide clinical decision-making and offers meaningful improvements in patient outcomes within these threshold ranges.

## Discussion

In this cross-sectional study involving elderly Asian patients with T2DM, the authors developed and internally validated a nomogram to predict the risk of sarcopenia based on demographic, clinical, and lifestyle factors. Key predictors identified were advanced age, male sex, poor glycemic control, hypoalbuminemia, hypothyroidism, alcohol history, and low physical activity. These findings highlight the complex, multifactorial nature of sarcopenia and support the utility of an integrative risk stratification approach.

These results align with existing evidence linking aging to sarcopenia, likely mediated by declines in anabolic hormones, oxidative stress, and chronic inflammation. These mechanisms reinforce the need for age-specific interventions to mitigate muscle loss.^12^^,^^15^ The observation that male gender independently increases sarcopenia risk may reflect sex-specific differences in muscle physiology, hormonal profiles (e.g., testosterone decline), and lifestyle factors such as physical activity levels.^16^^,^^17^ In the studied cohort, men demonstrated significantly lower physical activity levels compared to women, with a greater proportion reporting low physical activity (42.9 % vs. 35.4 %). This may contribute to the higher sarcopenia risk observed in males, aligning with evidence that physical inactivity exacerbates muscle loss, particularly in the context of T2DM. Unexpectedly, BMI did not differ significantly between groups (*p* = 0.764), possibly due to confounding by adiposity or specific sample characteristics. This warrants further investigation in larger, more diverse cohorts.

Among modifiable factors, poor glycemic control emerged as a strong predictor of sarcopenia. Elevated HbA1c levels, indicative of chronic hyperglycemia, have been implicated in several pathophysiological processes that damage muscle health. Hyperglycemia promotes the formation of Advanced Glycation End-products (AGEs), which disrupt muscle protein turnover, induce oxidative stress, and exacerbate mitochondrial dysfunction. Additionally, insulin resistance limits glucose uptake in muscle cells, resulting in energy deficits that impair protein synthesis and maintenance.^18^, ^19^, ^20^ These findings underscore the critical need for stringent glycemic management in elderly T2DM patients to reduce sarcopenia risk.

Nutritional status, assessed via serum albumin levels, was also identified as a significant predictor of sarcopenia. Low serum albumin, a marker of malnutrition and systemic inflammation, likely reflects insufficient dietary protein intake, which is essential for muscle maintenance. This finding is consistent with evidence suggesting that protein supplementation can enhance muscle health, particularly in older adults.^21^^,^^22^ Although vitamin D deficiency did not reach statistical significance in the present study (*p* = 0.072), it showed a marginal association with sarcopenia. This highlights the need for further investigation into the role of vitamin D in sarcopenia, especially considering its potential impact on muscle function.^23^^,^^24^ Similarly, hyperlipidemia demonstrated a trend toward association with sarcopenia (*p* = 0.169), suggesting a potential link that warrants further exploration.

Thyroid dysfunction, particularly hypothyroidism, was another significant risk factor in the present analysis. The impact of hypothyroidism on sarcopenia may result from metabolic dysregulation, reduced protein turnover, and impaired mitochondrial function.^25^^,^^26^ While the association between thyroid hormones and muscle health has been documented, the present findings suggest the need for routine screening and management of thyroid dysfunction in elderly T2DM patients.

Alcohol history also emerged as a significant independent predictor of sarcopenia in the studied cohort (OR = 2.53). These finding highlights alcohol consumption as a modifiable risk factor, particularly relevant for elderly patients with T2DM who are already vulnerable to muscle loss. Chronic alcohol use has been shown to impair muscle protein synthesis, disrupt anabolic signaling pathways (e.g., IGF-1/Akt/mTOR), and promote oxidative stress and mitochondrial dysfunction ‒ all of which contribute to muscle atrophy. These effects may be amplified in T2DM due to underlying insulin resistance and metabolic dysregulation. The present results align with previous studies linking alcohol intake to increased sarcopenia risk.^27^ Although the present study did not assess specific drinking patterns, the association observed suggests that even regular alcohol consumption may contribute to sarcopenia in this population.

In the present cohort, physical activity demonstrated a clear dose-response relationship with sarcopenia risk: individuals with moderate or high activity levels had substantially lower odds of sarcopenia compared to those with low activity. Supplemental analysis revealed that this relationship persisted even after adjusting for potential confounders such as BMI and comorbidities, further supporting the protective effect of physical activity. This aligns with prior evidence that an active lifestyle preserves muscle mass and function in older adults and may be especially beneficial in T2DM by enhancing glycemic control and muscle metabolism.^28^ Mechanistically, exercise increases muscle glucose uptake and insulin sensitivity ‒ limiting chronic hyperglycemia and the formation of damaging advanced glycation end‑products ‒ while also stimulating the release of myokines (e.g., irisin, IL‑6) that exert anti‑inflammatory and anabolic effects. Together, these processes protect against muscle protein degradation and counteract the pro‑inflammatory milieu of diabetes, making regular physical activity a practical strategy to mitigate sarcopenia in elderly T2DM patients.^29^

Clinically, the nomogram developed in this study provides a practical and accessible tool for the early identification of elderly T2DM patients at elevated risk of sarcopenia. By integrating both modifiable (e.g., glycemic control, nutritional status, thyroid function, physical activity) and non-modifiable (e.g., age, sex) factors, the model enables targeted, personalized interventions ‒ including optimized glycemic management, nutritional supplementation, thyroid monitoring, and structured exercise programs. This individualized approach holds promise for mitigating sarcopenia-related morbidity and improving functional outcomes in high-risk populations.^30^^,^^31^

The strengths of this study lie in its use of a well-characterized cohort, rigorous predictor selection via LASSO regression, and robust internal validation strategies, including calibration and decision curve analyses. However, several limitations must be acknowledged. First, the retrospective cross-sectional design limits causal inference and may introduce selection or reporting biases, despite the application of comprehensive statistical controls. Second, the study population was drawn from a geographically localized and ethnically homogeneous group, which may restrict the generalizability of findings. Future research should therefore prioritize multicenter, prospective studies encompassing diverse populations to validate and extend the nomogram’s applicability. Lastly, the absence of detailed data on antidiabetic pharmacotherapy precluded analysis of medication-specific effects on sarcopenia risk. Subsequent investigations should incorporate medication profiles to clarify how treatment regimens influence muscle mass and function in elderly T2DM cohorts.

## Conclusion

In summary, the authors have developed and internally validated a robust nomogram that integrates demographic, clinical, and lifestyle variables to accurately predict sarcopenia risk in elderly Asian patients with T2DM. By enabling early identification of high‑risk individuals, this tool supports personalized interventions ‒ such as optimized glycemic control, targeted nutritional strategies, and tailored exercise regimens ‒ to preserve muscle health and reduce disability. Prospective, multi‑center studies are now warranted to evaluate its implementation in routine care, assess cost‑effectiveness, and determine its impact on long‑term patient outcomes across diverse populations.

## Funding

No external funding was provided for this study.

## Declaration of competing interest

The authors declare that there are no conflicts of interest regarding the research, authorship, or publication of this manuscript.

## References

[1] Kim M., Won C.W. (2020). Sarcopenia in Korean community-dwelling adults aged 70 years and older: application of screening and diagnostic tools from the Asian Working Group for Sarcopenia 2019 update. J Am Med Dir Assoc.

[2] Cao M., Lian J., Lin X., Liu J., Chen C., Xu S. (2022). Prevalence of sarcopenia under different diagnostic criteria and the changes in muscle mass, muscle strength, and physical function with age in Chinese old adults. BMC Geriatr.

[3] Papadopoulou S.K. (2020). Sarcopenia: a contemporary health problem among older adult populations. Nutrients.

[4] Li R., Lin S., Tu J., Chen Y., Cheng B., Mo X. (2023). Establishment and evaluation of a novel practical tool for the diagnosis of pre-sarcopenia in young people with diabetes mellitus. J Transl Med.

[5] Chai K.C., Chen W.M., Chen M., Shia B.C., Wu S.Y. (2022). Association between preexisting sarcopenia and stroke in patients with type 2 diabetes mellitus. J Nutr Health Aging.

[6] Yang W., Wang J. (2024). Research progress on the pathogenesis and treatment of sarcopenia in elderly patients with diabetes. J Difficult Dis.

[7] Izzo A., Massimino E., Riccardi G., Della Pepa G. (2021). A narrative review on sarcopenia in type 2 diabetes mellitus: prevalence and associated factors. Nutrients.

[8] Anagnostis P., Gkekas N.K., Achilla C., Pananastasiou G., Taouxidou P., Mitsiou M. (2020). Type 2 Diabetes mellitus is associated with increased risk of sarcopenia: a systematic review and meta-analysis. Calcif Tissue Int.

[9] Gielen E., Dupont J., Dejaeger M., Laurent M.R. (2023). Sarcopenia, osteoporosis and frailty. Metabolism..

[10] Dennison E.M., Sayer A.A., Cooper C. (2017). Epidemiology of sarcopenia and insight into possible therapeutic targets. Nat Rev Rheumatol.

[11] Damluji A.A., Alfaraidhy M., AlHajri N., Rohant N.N., Kumar M., Al Malouf C. (2023). Sarcopenia and cardiovascular diseases. Circulation.

[12] van Dronkelaar C., Fultinga M., Hummel M., Kruizenga H., Weijs P.J.M., Tieland M. (2023). Minerals and sarcopenia in older adults: an updated systematic review. J Am Med Dir Assoc.

[13] American Diabetes Association (2021). Classification and diagnosis of diabetes: standards of medical care in diabetes ‒ 2021. Diabetes Care.

[14] Chen L.K., Woo J., Assantachai P., Auyeung T.-W., Chou M.-Y., Iijima K. (2020). Asian Working Group for Sarcopenia: 2019 consensus update on sarcopenia diagnosis and treatment. J Am Med Dir Assoc.

[15] Fielding R.A., Vellas B., Evans W.J., Bhasin S., Morley J.E., Newman A.B. (2011). Sarcopenia: an undiagnosed condition in older adults. Current consensus definition: prevalence, etiology, and consequences. International working group on sarcopenia. J Am Med Dir Assoc.

[16] Choe H.J., Cho B.L., Park Y.S., Roh E., Kim H.J., Lee S.-G. (2022). Gender differences in risk factors for the 2-year development of sarcopenia in community-dwelling older adults. J Cachexia Sarcopenia Muscle.

[17] Cho Y.J., Lim Y.H., Yun J.M., Yoon H.J., Park M. (2020). Sex- and age-specific effects of energy intake and physical activity on sarcopenia. Sci Rep.

[18] Kim H.U., Park S.P., Kim Y.K. (2021). Long-term HbA1c variability and the development and progression of diabetic retinopathy in subjects with type 2 diabetes. Sci Rep.

[19] Skovgaard D., Siersma V.D., Klausen S.B., Visnes H., Haukenes I., Bang C.W. (2021). Chronic hyperglycemia, hypercholesterolemia, and metabolic syndrome are associated with risk of tendon injury. Scand J Med Sci Sports.

[20] Brech G.C., Paula T.S., Fedele T.A., Dias A.S., Soares-Júnior J.M., Bordalo-Rodrigues M. (2020). Response to fatigue observed through magnetic resonance imaging on the quadriceps muscle in postmenopausal women. Clinics (Sao Paulo).

[21] Erdoğan K., Kara M., Şener F.E., Durmuş M.E., Çıtır Durmuşoğlu B.N., Abdulsalam A.J. (2025). Serum albumin as a biomarker of nutritional status in sarcopenia. J Bone Miner Metab.

[22] Zhang C., Zhang L., Zeng L., Wang Y., Chen L. (2024). Associations of serum albumin and dietary protein intake with all-cause mortality in community-dwelling older adults at risk of sarcopenia. Heliyon.

[23] Uchitomi R., Oyabu M., Kamei Y. (2020). Vitamin D and sarcopenia: potential of vitamin D supplementation in sarcopenia prevention and treatment. Nutrients.

[24] Prokopidis K., Giannos P., Katsikas Triantafyllidis K., Kechagias K.S., Mesinovic J., Witard O.C. (2022). Effect of vitamin D monotherapy on indices of sarcopenia in community-dwelling older adults: a systematic review and meta-analysis. J Cachexia Sarcopenia Muscle.

[25] Hu X., Zhang L., Zhang M., Mi W., Sun Y., Wang Y. (2023). Correlation of subclinical hypothyroidism with sarcopenia and its components in the Chinese older adults. Endocrine.

[26] Szlejf C., Suemoto C.K., Janovsky C.C.P.S., Barreto S.M., Diniz M.F.H.S., Lotufo P.A. (2020). Thyroid function and sarcopenia: results from the ELSA-Brasil study. J Am Geriatr Soc.

[27] Yoo J.-I., Ha Y.-C., Lee Y.-K., Hana-Choi Yoo M-J, Koo K.-H (2017). High prevalence of sarcopenia among binge drinking elderly women: a nationwide population-based study. BMC Geriatr.

[28] Wen C.Y., Lien A.S., Jiang Y.D (2022). Sarcopenia in elderly diabetes. J Diabetes Investig.

[29] Argyropoulou D., Geladas N.D., Nomikos T., Paschalis V. (2022). Exercise and nutrition strategies for combating sarcopenia and type 2 diabetes mellitus in older adults. J Funct Morphol Kinesiol.

[30] Sayer A.A., Cruz-Jentoft A. (2022). Sarcopenia definition, diagnosis and treatment: consensus is growing. Age Ageing.

[31] Coletta G., Phillips S.M. (2023). An elusive consensus definition of sarcopenia impedes research and clinical treatment: a narrative review. Ageing Res Rev.

